# Repeated Cross-Sectional Surveillance and ORF5-Based Molecular Epidemiology of Porcine Reproductive and Respiratory Syndrome Virus in Anhui Province, China, 2019–2024

**DOI:** 10.3390/ani16172672

**Published:** 2026-08-25

**Authors:** Hua Liu, Zhao Qi, Yingchun Zhou, Wei Wang, Lixia Wang, Yan Shen, Chang Liu, Dongdong Yin, Zhenzhen Chai, Qianqian Duan, Jun Wang, Qian Wang, Xi Chen, Changsheng He, Songhe Zhan, Liangqiang Zhu, Kezong Qi

**Affiliations:** 1 College of Veterinary Medicine, Anhui Agricultural University, Hefei 230036, China; vetliuhua@163.com (H.L.); qizhao1050@ahau.edu.cn (Z.Q.); 2 Anhui Center for Animal Disease Prevention and Control, Hefei 230091, China; zhouzhou1220@126.com (Y.Z.); 18956042178@163.com (W.W.); yshen2009@163.com (Y.S.); 20720466@stu.ahau.edu.cn (C.L.); 15385887631@163.com (Z.C.); vetdqq@126.com (Q.D.); wangjun101019@126.com (J.W.); xianrenzhange23@126.com (Q.W.); chenxi7856@163.com (X.C.); changshenghe@126.com (C.H.); vetzhu@126.com (L.Z.); 3 Anhui Provincial Key Laboratory of Veterinary Pathobiology and Disease Prevention and Control, Hefei 230036, China; 4 Anhui Hongqiao Zhongke Gene Technology Co., Ltd., Hefei 231131, China; wang.lixia@sslab.com.cn; 5 Institute of Animal Husbandry and Veterinary Medicine, Anhui Academy of Agricultural Sciences, Hefei 230031, China; ydd@aaas.org.cn

**Keywords:** porcine reproductive and respiratory syndrome virus, cross-sectional surveillance, pooled-sample testing, *ORF5* gene, molecular epidemiology, NADC30-like, NADC34-like

## Abstract

Porcine reproductive and respiratory syndrome virus (PRRSV) remains a major threat to swine production, and its circulation after the African swine fever outbreak requires continued surveillance. This study investigated the temporal, regional, and genetic characteristics of PRRSV in Anhui Province from September 2019 to November 2024. Ten rounds of repeated cross-sectional surveillance were conducted at 147 slaughterhouses and 21 rendering plants. Tissue samples were collected by random, cluster, or risk-based sampling, pooled in groups of five, and tested by RT-qPCR. Representative positive samples with Ct values < 25 underwent ORF5 amplification, Sanger sequencing, and phylogenetic analysis. A total of 994 site visits yielded 18,733 tissue samples. No positive samples were detected in autumn 2020 or spring 2021, whereas PRRSV was detected again from autumn 2021 and subsequently fluctuated. The highest site-level positivity was 41.18% in autumn 2023, and the highest estimated individual-level positivity was 4.66% in spring 2023. Regional differences were statistically significant, with the highest site-level positivity in northern Anhui, and pooled-sample positivity was higher in rendering plants than in slaughterhouses. All 24 ORF5 sequences belonged to PRRSV-2 and mainly clustered with NADC30-like, NADC34-like, or MLV/classical strains. These findings demonstrate temporal fluctuations and lineage coexistence, supporting continued multisource surveillance and broader genomic and antigenic evaluation.

## Simple Summary

Porcine reproductive and respiratory syndrome is a major disease of pigs that can cause reproductive failure, breathing problems, poor growth, and substantial economic losses. This study examined how the virus changed over time and across regions of Anhui Province after the African swine fever outbreak. From September 2019 to November 2024, researchers carried out 10 rounds of surveillance at 147 slaughterhouses and 21 facilities that process dead pigs. In total, 18,733 tissue samples were tested for viral genetic material. No positive samples were found in autumn 2020 or spring 2021, but the virus was detected again from autumn 2021 onward and then fluctuated over time. Detection levels were generally higher in northern Anhui, and the overall difference among the three regions was statistically significant. Samples from dead pigs were more likely to contain the virus than samples from slaughter pigs. Comparison of the genetic material from 24 virus samples showed that several distinct virus groups were circulating at the same time, with newer groups becoming more common after 2021. These findings support continued surveillance across farms, slaughterhouses, and rendering plants, together with broader genetic testing and improved evaluation of whether vaccines match locally circulating viruses.

## 1. Introduction

Porcine reproductive and respiratory syndrome (PRRS) is one of the most economically important viral diseases affecting the global swine industry. Its causative agent, porcine reproductive and respiratory syndrome virus (PRRSV), exhibits high genetic variability and frequent recombination, facilitating persistent infection and endemic circulation. Infection can cause reproductive failure in sows, respiratory disease in piglets, and impaired growth performance, resulting in substantial economic losses [1,2,3,4,5]. In China, PRRSV-2 predominates, with lineages 1, 3, 5, and 8 currently circulating. In recent years, lineage 1 NADC30-like and NADC34-like strains have been increasingly detected, while the coexistence and recombination of multiple lineages have further complicated viral epidemiology [6,7,8,9]. Under the updated ORF5-based classification framework, PRRSV-2 strains are assigned to lineages 1, 3, 5, 8, and 9, with NADC30-like and NADC34-like strains belonging to different sublineages of lineage 1 [10]. Given the marked genetic and antigenic differences among lineages, continuous surveillance of lineage shifts and regional transmission patterns remains essential for targeted PRRS prevention and control [11].

Following the African swine fever outbreak in 2018, major changes occurred in China’s swine production systems, biosecurity practices, and interregional animal movement. Nationwide genomic surveillance suggested that the outbreak may have affected the recombination frequency of PRRSV-2 without substantially altering its dominant lineage composition [12]. However, previous PRRSV studies in Anhui Province have mainly focused on clinical cases or individual production stages, with limited long-term surveillance covering both slaughterhouses and rendering plants. Slaughterhouse surveillance reflects PRRSV detection in apparently healthy slaughter pigs and supports regional trend monitoring, whereas rendering plants receive dead pigs and may increase the likelihood of detecting clinically relevant viruses; surveillance combining both sources therefore provides complementary epidemiological information. Therefore, repeated cross-sectional surveillance was conducted from September 2019 to November 2024. Pooled-sample RT-qPCR was used to assess the temporal, regional, and site-specific distribution of PRRSV, and representative positive samples were subjected to ORF5-based phylogenetic analysis, an approach commonly used in PRRSV molecular epidemiological surveillance [13]. This study aimed to provide epidemiological evidence for risk surveillance and differentiated PRRS control in Anhui Province.

## 2. Materials and Methods

### 2.1. Study Design and Target Populations

This study employed a repeated cross-sectional design and included two target populations: (1) slaughter pigs originating from Anhui Province that passed entry inspection at local slaughterhouses, and (2) dead pigs collected from farms or households within the province and transported to rendering plants.

The surveillance framework comprised 147 operational slaughterhouses, representing 98.7% of all such facilities in the province, and 21 rendering plants, achieving 100% coverage. The analytical unit was defined as a “site–sampling period” combination. Because the same site could be repeatedly included during different sampling periods, each “site visit” referred to an independent sampling event.

**Figure 1:**
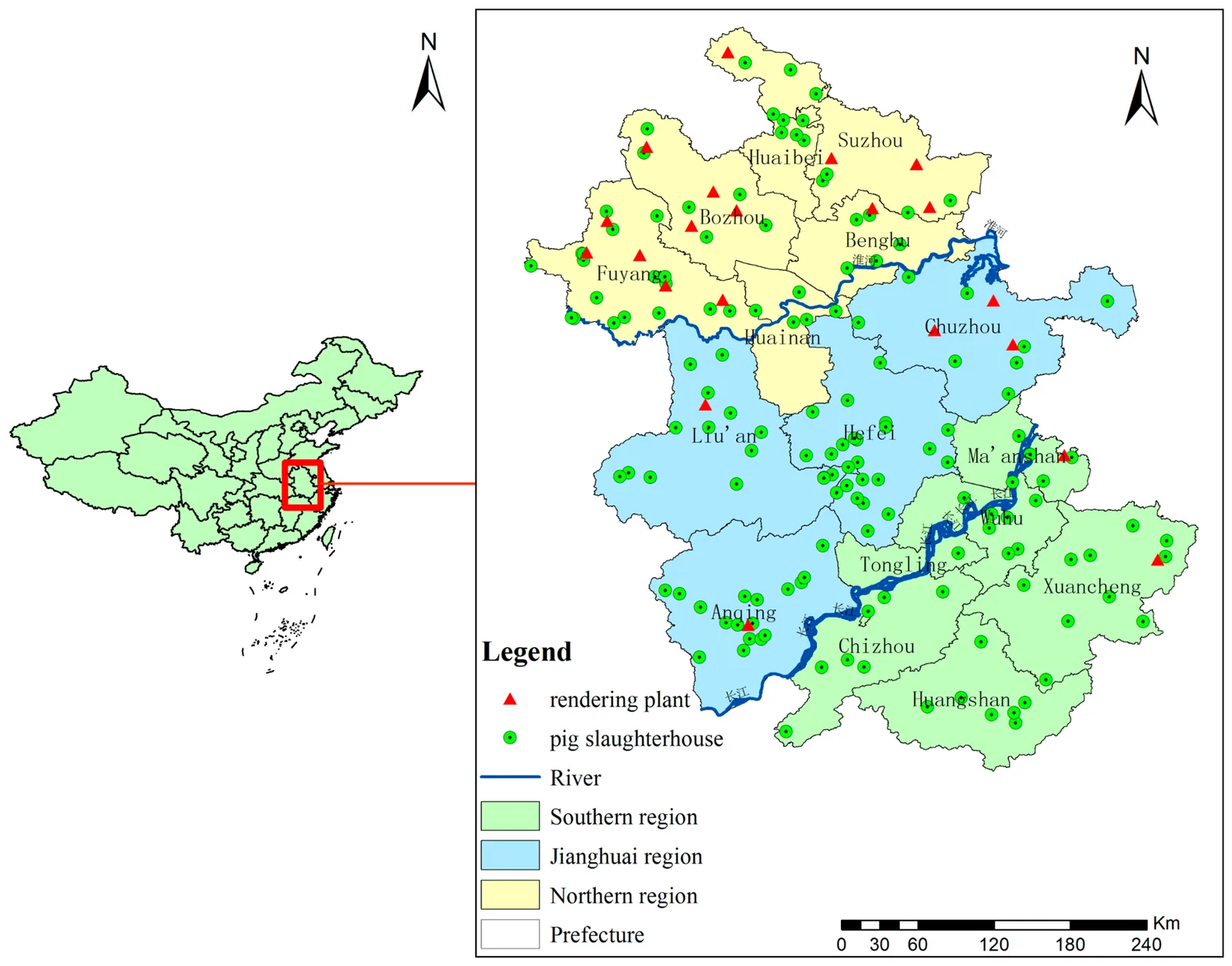
Distribution of pig slaughterhouses and rendering plants in Anhui Province, China. The map was created using ArcGIS 10.1 (Esri, Redlands, CA, USA).

To assess regional differences, sampling locations were categorized into northern Anhui, the Jianghuai region, and southern Anhui according to the conventional administrative and geographical division of Anhui Province (i.e., regions north of the Huai River, between the Huai River and the Yangtze River, and south of the Yangtze River, respectively); the cities assigned to each region are listed in Section 2.7.2. The geographical distribution of the surveillance sites is shown in Figure 1.

### 2.2. Sampling Strategy and Sample Size

#### 2.2.1. Surveillance Sites and Sampling Periods

In principle, two rounds of centralized sampling were conducted annually: one in spring (April–June) and one in autumn (September–November). Spring sampling was not conducted in 2020 because of COVID-19 pandemic control measures that restricted interregional movement and fieldwork. For slaughterhouses, simple random sampling was used from 2019 to 2021, whereas cluster sampling was adopted from 2022 to 2024 according to facility operating conditions. Rendering plants were sampled using cluster sampling from 2021 to 2024. During the simple random sampling period, the required number of surveillance sites was calculated using the sample-size formula for estimating a population proportion (Equation (1)).

(1) $${N=z}^{2}\times P\times \left(1\;-\;P\right)÷e^{2}$$

where (N) is the required number of sites, (P) is the expected prevalence (set at 20% in 2019 and at 20%, 30%, and 30% for the three sampling periods of 2020–2021, respectively), (z) is the standard normal quantile (z = 1.96 for a 95% confidence level), and (e) is the allowable margin of error (set at 12% in 2019 and at 12%, 12%, and 10% for the three sampling periods of 2020–2021, respectively), with minor adjustments according to the operational circumstances of the slaughterhouses.

#### 2.2.2. Individual-Level Sampling and Sample Size

(1) Slaughterhouses: Pig batches were numbered according to their source, and mandibular lymph nodes were collected along the slaughter line using systematic sampling at fixed intervals. Assuming an expected individual-level positivity rate of 50%, a 20% margin of error, and a 95% confidence level, 10–30 pigs were sampled from each site during each sampling period.

(2) Rendering plants: A disease-detection sampling framework was used, assuming a diagnostic sensitivity (Se) of 99.99%. The minimum individual-level sample size was estimated using Equation (2) [14]:

(2) $$n\;=\;\left[1\;-\;\;{\left(1\;-\;\;\mathrm{CL}\right)}^{\frac{1}{D}}\right]\times \left(N’\;-\;\;\frac{D\;-\;\;1}{2}\right)$$

where (CL) is the confidence level, set at 95%; (N’) is the number of dead pigs processed daily; and (D) is the expected number of infected animals. Based on an average daily throughput of 50 pigs and D = 25, the minimum sample size was five pigs per sampling event.

In practice, risk-based sampling was applied, prioritizing pigs with premortem fever or respiratory signs, obvious lesions, or an unknown cause of death. Lung, spleen, lymph node, and other affected tissues were aseptically collected, with inguinal lymph nodes preferentially sampled from larger animals. Because risk-based sampling was used, the observed positivity rate represents the detection level among the selected dead pigs and should not be extrapolated directly to all dead pigs or the general farm population.

#### 2.2.3. Pooled-Sample Testing Strategy

To improve testing efficiency, five tissue samples, each collected from a different individual pig of the same farm or source batch, were combined in approximately equal amounts to form one sample pool. Individual samples within a positive pool were not retested separately.

The sensitivity (Se) and specificity (Sp) of the test kit were both 99.99%. The herd-level sensitivity (HSe) and herd-level specificity (HSp) of the pooled testing strategy were calculated using Equations (3) and (4), with the expected herd prevalence (P) set at 2–50% based on empirical values:

(3) $$HSe=1-{\left\{\left[1-{\left(1-P\right)}^{m}\right]\times \left(1-PSe\right)+{\left(1-P\right)}^{m}\times PSp\right\}}^{r}$$

(4) $${HSp=Sp}^{m}$$

where HSe and HSp are the herd-level sensitivity and herd-level specificity of the pooled testing strategy, respectively; P is the expected herd prevalence; m is the number of samples in each pool; and r is the number of pools.

Therefore, the individual-level positivity rate was estimated using a fixed-pool-size model, assuming near-perfect diagnostic sensitivity and specificity, as supported by Equations (3) and (4). Pooling was performed within each source batch; therefore, when the number of samples from a batch was not a multiple of five, the final pool of that batch contained fewer than five samples.

### 2.3. Sample Collection, Transportation, and Storage

From September 2019 to November 2024, 10 surveillance rounds comprising 994 site visits were completed, including 836 visits to slaughterhouses and 158 visits to rendering plants. A total of 18,733 tissue samples, including lymph nodes, were collected (Table 1). No sampling was conducted in spring 2020 due to restrictions imposed in response to the COVID-19 pandemic.

Samples were collected using disposable sterile instruments. Tissue samples from each pig were individually packaged, labeled, and immediately placed in a refrigerated container at 4 °C. Sampling records included the date, location, site identification number, pig source, ear-tag number, and clinical information. Samples were delivered to local laboratories within 6 h and temporarily stored at −20 °C for no more than 24 h. After sampling within each region was completed, the samples were transported to the testing laboratory under a −20 °C cold chain and stored at −80 °C until analysis.

### 2.4. Major Instruments and Reagents

The principal instruments included a KingFisher Flex automated magnetic-bead nucleic acid purification system (Thermo Fisher Scientific Oy, Waltham, MA, USA) and CFX96 Touch and CFX Opus 96 real-time PCR systems (Bio-Rad Laboratories, Inc., Hercules, CA, USA). The magnetic-bead viral DNA/RNA extraction kit was obtained from Nanjing Zoonbio Medical Technology Co., Ltd. (Nanjing, China), and the commercial PRRSV real-time RT-PCR detection kits, which target the PRRSV-1 N gene and the PRRSV-2 M gene, were purchased from Qingdao Lijian Bio-Tech Co., Ltd. (Qingdao, China).

The primer and probe sequences were designed based on the conserved regions of the ORF6/ORF7 sequences of the European strain in GenBank. The primer sequences are as follows:

Forward primer P1: 5′-CAGATGCAGAYTGTGTTGCCT-3′;

Reverse primer P2: 5′-TGGAGDCCTGCAGCACTTTC-3′;

Probe sequences P3: 5′-(FAM) ATACATTCTGGCCCCTGCCCAYCACGT(TAMRA)-3′.

The primer and probe sequences were designed based on the conserved regions of ORF7 and 3′UTR from the American strain in GenBank. The primer sequences are as follows:

Upstream primer P1: 5′-GCACTGATTGACAYTGTGCC-3′;

Downstream primer P2: 5′-CGCATGGTTCTCGCCAAT-3′;

Probe Sequence P3: 5′-(FAM) AGTCACCTATTCAATTAGGGCGACCG(TAMRA)-3′.

### 2.5. Sample Processing and Nucleic Acid Extraction

Tissue samples were partially thawed at 4 °C. In a biosafety cabinet, approximately 50–100 mg of each sample was transferred to a grinding tube containing 1.5 mL of PBS and three sterile steel beads, and each sample was homogenized individually at 60 Hz for 55 s, followed by a 5 s interval, for three to five cycles. The homogenates were centrifuged at 13,000× *g* for 2 min at 4 °C, and equal volumes of the supernatants from five samples of the same source were then combined to form one pool. All sample-processing steps were performed at 4 °C, and processed samples were aliquoted and stored at −80 °C to minimize nucleic acid degradation. Subsequently, 200 μL of the supernatant was collected, and total nucleic acids were extracted using the KingFisher Flex system according to the manufacturer’s instructions and stored at −80 °C until analysis.

### 2.6. RT-qPCR Detection

RT-qPCR was performed in a total reaction volume of 25 μL containing 5 μL of extracted nucleic acid. Positive and negative controls were included in each run. The cycling conditions were as follows: reverse transcription at 50 °C for 5 min, initial denaturation at 95 °C for 30 s, followed by 40 cycles of 95 °C for 10 s and 60 °C for 30 s. FAM fluorescence was recorded during the 60 °C step. When the controls met the validity criteria, samples with a Ct value < 37 (the cutoff recommended in the kit manufacturer’s instructions) and a typical sigmoidal amplification curve were considered PRRSV-positive.

### 2.7. Epidemiological and Statistical Analyses

#### 2.7.1. Estimation of Herd and Individual-Level Positivity Rates

A site was classified as PRRSV-positive if at least one sample pool tested positive by RT-qPCR. The herd apparent prevalence (HAp) was calculated as the number of positive sites divided by the total number of sampled sites using Equation (5):

HAp (%) = Number of positive sites/Number of sampled sites × 100%(5)

For the simple random sampling period, the herd true prevalence (HTp) was estimated using Equation (6), and the corresponding 95% confidence interval (CI) was estimated using Equation (7).

(6) $$HTP=\left(HAp+HSp-1\right)÷\left(HSe+HSp-1\right)$$

(7) $$95\%CI=HTP\pm 1.96\times \sqrt{HTP\times \left(1-HTP\right)÷N}$$

Individual prevalence and exact 95% CIs were estimated using the “Pooled Prevalence Estimates for Perfect Tests with Exact Confidence Limits” module in Ausvet EpiTools (https://epitools.ausvet.com.au/?page=PPFreq1) (accessed on 9 July 2026). The pool size (five samples), total number of pools tested, and number of positive pools were entered into the model. The estimation assumed a fixed pool size, independence among samples, and near-perfect diagnostic sensitivity and specificity.

#### 2.7.2. Comparisons by Sampling Period, Region, and Site Type

Temporal differences in site-level and pooled-sample positivity rates were compared across sampling periods. For regional analyses, the three geographical regions were compared. Comparisons between slaughterhouses and rendering plants included data collected from autumn 2021 to autumn 2024. Count data—including temporal, regional, and site-type comparisons—were analyzed using the chi-square test or Fisher’s exact test when any expected cell count was <5. All tests were two-sided, and *p* < 0.05 was considered statistically significant. Statistical analyses were performed in R (version 4.3.2; R Foundation for Statistical Computing, Vienna, Austria).

### 2.8. ORF5 Amplification, Sequencing, and Phylogenetic Analysis

Representative positive samples with Ct values < 25 were selected for Sanger sequencing according to sampling period, geographical region, and Ct value. The primers used for ORF5 amplification were as follows:

ORF5-F: 5′-GGCAATGTGTCAGGCATCGTGGCTG-3′

ORF5-R: 5′-GGCCAGAATGTACTTGCGGCCTAGC-3′

The primers were designed in-house based on conserved regions flanking the ORF5 gene of PRRSV-2 reference sequences retrieved from GenBank. An 1116 bp fragment containing ORF5 was amplified, and the resulting sequences were assembled and manually edited using SeqMan (DNASTAR 7.1, Madison, WI, USA). The edited sequences were aligned with representative PRRSV reference sequences retrieved from GenBank using MEGA 12. Reference sequences were selected to represent the major PRRSV-2 lineages and sublineages currently circulating in China, including NADC30-like, NADC34-like, classical/highly pathogenic, and VR-2332/MLV-related strains, following the updated ORF5-based lineage classification framework [10]; their GenBank accession numbers are listed in Table S1. Phylogenetic trees were constructed using the neighbor-joining method with the Kimura 2-parameter substitution model in MEGA 12, and branch reliability was assessed by bootstrap analysis with 1000 replicates. Viral lineages were assigned according to their clustering relationships with established reference strains [10].

## 3. Results

### 3.1. Temporal Distribution

Significant overall temporal differences (omnibus chi-square tests across the 10 surveillance rounds) were observed in both site-level and pooled-sample positivity rates (site level: χ^2^ = 83.04, df = 9, *p* < 0.001; pooled samples: χ^2^ = 153.04, df = 9, *p* < 0.001; Table 2). All expected cell counts exceeded 5, supporting the use of the chi-square test despite zero observed counts in two periods, and the results were unchanged when the two zero-detection periods were excluded. No pairwise post hoc comparisons were performed; the period-specific patterns described below are therefore intended as descriptive observations. The site-level positivity rate was 40.00% in autumn 2019, whereas no positive samples were detected in autumn 2020 or spring 2021. After PRRSV was detected again in autumn 2021, site-level positivity rates fluctuated between 17.45% and 41.18%, peaking in autumn 2023. From autumn 2021 onward, the estimated individual-level positivity rate ranged from 1.76% to 4.66%, with the highest value observed in spring 2023.

### 3.2. Regional Distribution

**Figure 2:**
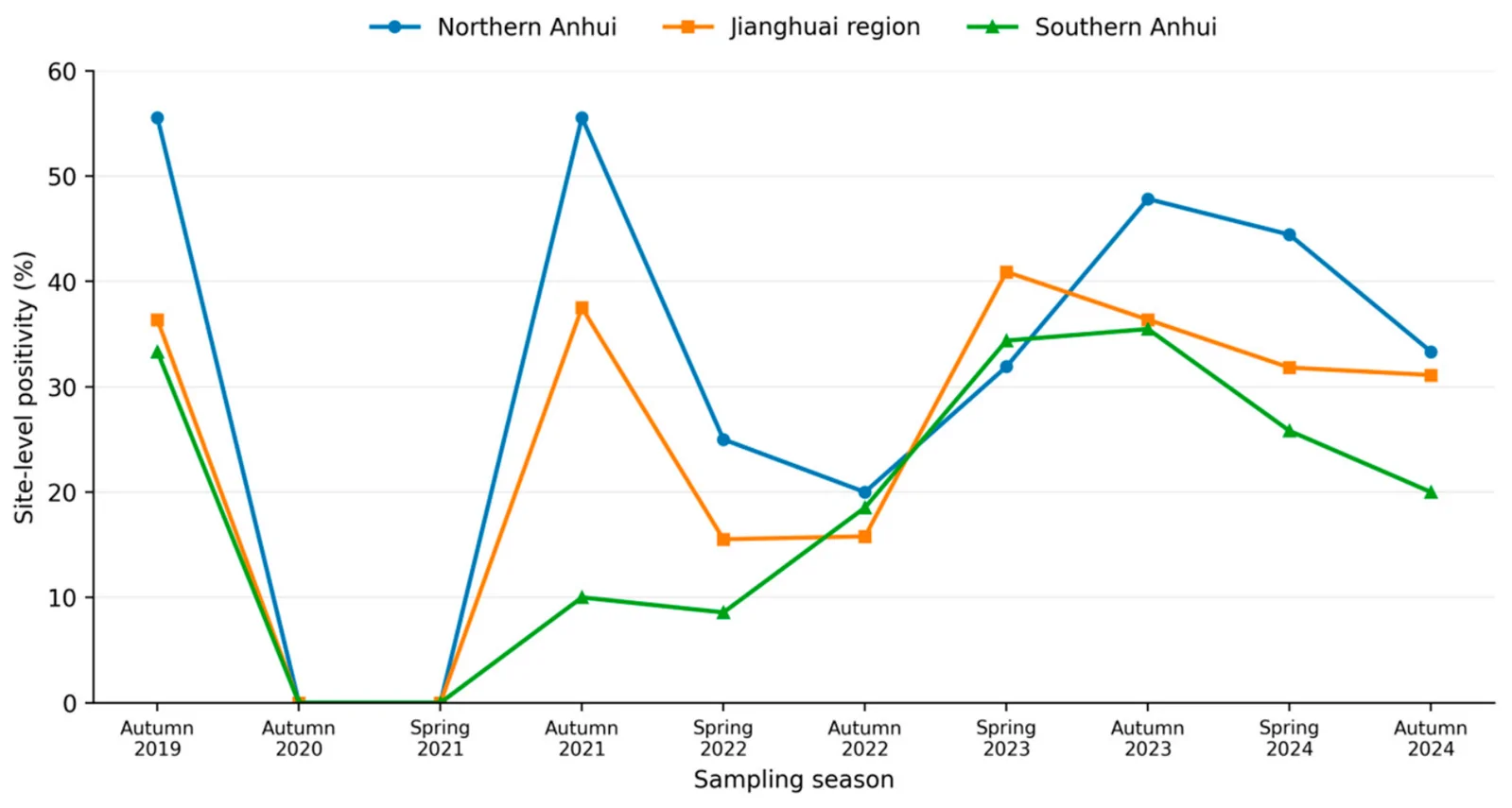
Site-level PRRSV positivity rates by region in Anhui Province, 2019–2024.

Overall, the site-level positivity rate was higher in northern Anhui than in the Jianghuai and southern Anhui regions, and the overall difference among the three regions was statistically significant (χ^2^ = 10.663, df = 2, *p* < 0.005). No positive samples were detected in any region in autumn 2020 or spring 2021. Thereafter, positivity rates fluctuated across all three regions. Northern Anhui showed relatively high positivity rates during several sampling periods, whereas fluctuations were less pronounced in the Jianghuai and southern Anhui regions (Figure 2). Because the number of sites sampled varied among regions and sampling periods, these period-specific patterns were interpreted descriptively.

### 3.3. PRRSV Detection by Site Type

From autumn 2021 to autumn 2024, site-level positivity rates ranged from 16.28% to 42.42% in slaughterhouses and from 19.05% to 47.06% in rendering plants (Table 3). When data from all sampling periods were combined, the site-level positivity rates were 28.94% (204/705) and 31.21% (44/141), respectively, with no significant difference between the two site types (χ^2^ = 0.19, *p* = 0.661). In contrast, the pooled-sample positivity rate was significantly higher in rendering plants than in slaughterhouses [28.74% (48/167) vs. 12.12% (327/2698); χ^2^ = 36.75, *p* < 0.001]. The estimated individual-level positivity rates ranged from 5.03% to 9.10% in rendering plants and from 1.52% to 4.40% in slaughterhouses.

### 3.4. Phylogenetic Characteristics of ORF5 Sequences

**Figure 3:**
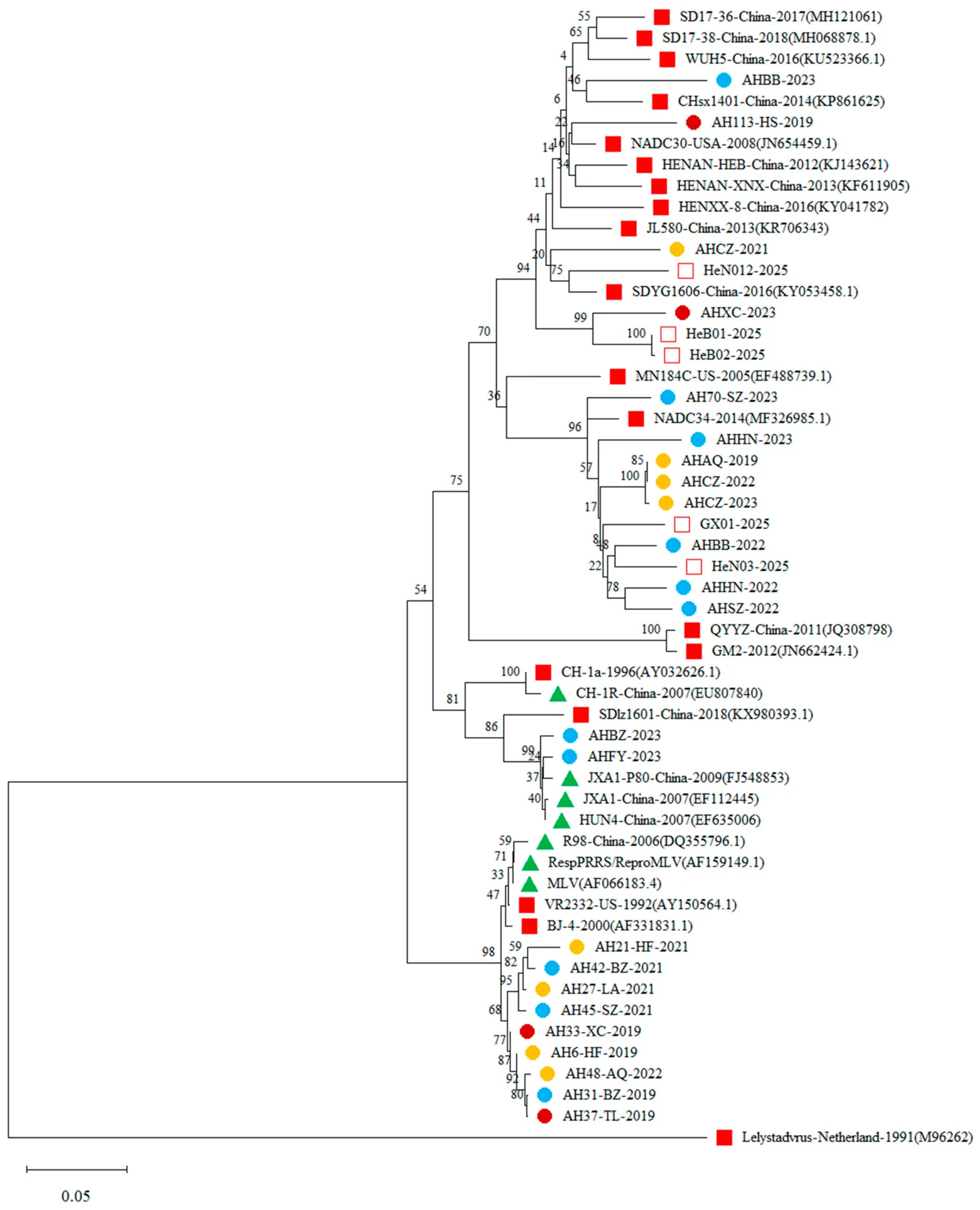
Phylogenetic tree of porcine reproductive and respiratory syndrome virus based on ORF5 nucleotide sequences. The phylogenetic tree was constructed in MEGA version 12.0 using the Neighbor-Joining method under the Kimura 2-parameter model. Bootstrap values (%) derived from 1000 replicates are shown at the corresponding nodes: ▲ indicate reference vaccine strains; ■ indicate reference circulating strains; □ indicate sequences from other regions; ● indicate sequences from northern Anhui; ● indicate sequences from the Jianghuai region; ● indicate sequences from southern Anhui.

A total of 24 PRRSV ORF5 sequences were obtained from Anhui Province. Phylogenetic analysis classified all sequences as PRRSV-2 and identified three major clusters: lineage 1 (L1), represented by NADC30-like and NADC34-like strains; lineage 8 (L8), associated with classical or highly pathogenic strains such as CH-1a and JXA1; and lineage 5 (L5), related to VR-2332 and modified live vaccine strains (Figure 3).

Temporally, several sequences obtained during 2019–2021 clustered with MLV-related or classical reference strains. After 2021, most sequences grouped within NADC30-like or NADC34-like branches. Bootstrap support was generally high (mostly ≥70%) for the deep nodes defining the major lineage-level clusters, whereas support for terminal (strain-level) nodes varied widely and was frequently below 50%; therefore, within-lineage relationships should be interpreted with caution. Because only the ORF5 region was analyzed, these findings describe genetic relationships and should not be interpreted as evidence of whole-genome recombination.

Geographically, sequences from the Jianghuai region were tentatively grouped with the NADC34-like and MLV-related branches, and several sequences obtained during 2022–2023 were closely related to the NADC34 reference strain. Only a limited number of sequences were obtained from southern Anhui, all of which clustered within the NADC30-like branch. These regional patterns may have been influenced by differences in the number of positive samples and the criteria used for sequencing selection. These regional and temporal patterns are descriptive and tentative: with only 24 sequences spanning six years and three regions, the low bootstrap support of many terminal nodes indicates limited phylogenetic resolution, and the geographic interpretations above should therefore be treated with caution.

Overall, the PRRSV sequences obtained through surveillance in Anhui Province showed the coexistence of NADC30-like, NADC34-like, and MLV/classical strain-related lineages, with an increasing proportion of L1-related sequences after 2021. Some local sequences were genetically distant from commonly used vaccine reference strains in the ORF5 phylogenetic tree. However, this finding only supports the need for further antigenic matching and cross-protection studies and cannot, by itself, demonstrate immune escape or vaccine failure.

## 4. Discussion

This study revealed that PRRSV detection in Anhui Province did not remain stable from 2019 to 2024 but followed a phased pattern of detection, non-detection, re-detection, and subsequent fluctuation. The absence of positive samples in autumn 2020 and spring 2021 only reflects the samples examined during those periods and should not be interpreted as evidence that PRRSV had disappeared from the province. Enhanced biosecurity, changes in swine population structure, and altered animal movement patterns after the African swine fever outbreak may have temporarily reduced opportunities for viral transmission. Nationwide genomic surveillance likewise suggested that the outbreak may have affected PRRSV-2 recombination frequency without fundamentally changing the dominant lineage composition [12]. The renewed detection after autumn 2021 may have been associated with the accumulation of susceptible pigs, increased animal movement, and the spread of L1-related strains. However, the repeated cross-sectional design of this study does not permit direct causal inference.

Most ORF5 sequences obtained in this study clustered within NADC30-like and NADC34-like branches, consistent with the increasing proportion of lineage 1 PRRSV-2 strains among detected sequences in China [7,15,16,17,18]. Under the updated ORF5 classification framework, Chinese PRRSV-2 strains are assigned to lineages L1, L3, L5, L8, and L9, with NADC30-like and NADC34-like strains belonging to different L1 sublineages [10]. Sequences from northern Anhui were distributed across several branches, possibly reflecting extensive interregional pig movement and diverse sample origins. Several sequences from the Jianghuai region were closely related to NADC34-like reference strains, whereas only a few NADC30-like sequences were obtained from southern Anhui. Because the number of sequenced samples was uneven among regions, these findings cannot be used to estimate regional lineage proportions precisely. Recent reports of PRRSV-1 and recombinant PRRSV-2 strains involving multiple lineages in Anhui further indicate that the regional viral population may be more complex than suggested by ORF5 analysis alone [19,20,21].

Slaughterhouse samples provide information on PRRSV nucleic acid detection among apparently healthy pigs entering the slaughter chain and are therefore useful for regional trend surveillance. In contrast, rendering plants receive dead pigs and may increase the likelihood of detecting clinically relevant viruses. Among the selected samples examined in this study, pooled-sample positivity was significantly higher in rendering plants than in slaughterhouses, whereas site-level positivity did not differ significantly between the two facility types. This difference should be interpreted cautiously because risk-based sampling at rendering plants and differences in the types of tissues collected may have influenced the comparison. Therefore, this observation should not be extrapolated to population-level PRRSV prevalence or interpreted as evidence that PRRSV infection caused death.

Some local ORF5 sequences were genetically distant from vaccine reference strains such as CH-1R, JXA1-P80, and MLV, highlighting the need to evaluate the compatibility between the vaccine and circulating strains [22,23]. Nevertheless, ORF5 genetic distance is not equivalent to antigenic distance and cannot independently predict clinical protection. Vaccine selection should therefore be based on neutralization and challenge studies, farm infection status, and vaccination programs. A more prudent control strategy is to continuously monitor dominant lineage changes, avoid unstructured alternation among different modified live vaccines, and strengthen quarantine of introduced pigs, acclimatization of replacement pigs, batch production, and farm biosecurity [24].

Several limitations should be acknowledged. First, sampling designs differed across years and facility types—in particular, slaughterhouse sampling changed from simple random sampling (2019–2021) to cluster sampling (2022–2024), which may have introduced systematic differences in site selection despite an essentially unchanged sampling frame—and repeatedly sampled sites were not analyzed using longitudinal models; therefore, temporal comparisons mainly represent descriptive trends. Second, risk-based sampling at rendering plants introduced potential selection bias. Third, individuals within positive pools were not retested, meaning that individual-level positivity estimates depended on model assumptions; in addition, the assumed independence among samples may have been violated by within-batch clustering of pigs sharing transport or housing, and the effect of pooling-related dilution on diagnostic sensitivity was not independently evaluated. Fourth, only 24 samples underwent ORF5 sequencing; the small sequence dataset, the low bootstrap support of many terminal nodes, and the known inability of distance-based phylogenetic methods to resolve clades reliably in the presence of recombination substantially limit the resolution of within-lineage relationships and any conclusions drawn from them, including the ability to identify whole-genome recombination, virulence characteristics, or vaccine cross-protection. In addition, the experimental design did not include testing for other co-circulating pathogens or serological assays, and pooled-sample testing without individual retesting precluded direct measurement of individual-level prevalence. Future studies should expand whole-genome sequencing of low-Ct samples, deposit sequences in public databases, and integrate virus isolation, serological testing, and farm production data for further validation.

## 5. Conclusions

Repeated cross-sectional surveillance at slaughterhouses and rendering plants in Anhui Province from 2019 to 2024 revealed marked temporal fluctuations and some regional heterogeneity in PRRSV detection. All 24 ORF5 sequences were classified as PRRSV-2, with the coexistence of NADC30-like, NADC34-like, and MLV/classical strain-related lineages. L1-related sequences became predominant after 2021. Establishing a continuous, integrated surveillance system linking multiple stages, together with whole-genome sequencing of representative samples and evaluation of antigenic matching between circulating and vaccine strains, is recommended to support risk stratification and targeted PRRS control in Anhui Province.

## Figures and Tables

**Table 1:** Number of site visits and tissue samples collected for PRRSV surveillance in Anhui Province, 2019–2024.

Sampling Period	Slaughterhouses		Rendering Plants	
	Site Visits	Samples	Site Visits	Samples
Autumn 2019	35	1229	0	0
Autumn 2020	41	1413	0	0
Spring 2021	55	1650	17	190
Autumn 2021	54	1635	17	210
Spring 2022	129	1966	20	100
Autumn 2022	123	1851	21	110
Spring 2023	102	2040	21	105
Autumn 2023	99	1970	20	100
Spring 2024	99	1968	21	106
Autumn 2024	99	1975	21	115
Total	836	17,697	158	1036

**Table 2:** Site-level positivity rates and estimated individual-level PRRSV positivity rates in Anhui Province, 2019–2024.

Sampling Period	Positive/Total Sites	HAp, % (95% CI)	Positive/Total Pools	Estimated Individual-Level Positivity, % (95% CI)
Autumn 2019	14/35	40.00 (25.55–56.43)	35/246	3.02 (2.11–4.18)
Autumn 2020	0/41	0.00 (0.00–8.57)	0/277	0.00 (0.00–0.27)
Spring 2021	0/72	0.00 (0.00–5.07)	0/364	0.00 (0.00–0.20)
Autumn 2021	26/71	36.62 (26.37–48.24)	48/369	2.75 (2.03–3.63)
Spring 2022	26/149	17.45 (12.20–24.34)	43/409	2.20 (1.59–2.95)
Autumn 2022	26/144	18.06 (12.63–25.14)	35/411	1.76 (1.23–2.45)
Spring 2023	44/123	35.77 (27.85–44.56)	91/429	4.66 (3.76–5.69)
Autumn 2023	49/119	41.18 (32.74–50.16)	49/414	2.49 (1.84–3.28)
Spring 2024	42/120	35.00 (27.05–43.88)	36/415	1.80 (1.26–2.48)
Autumn 2024	35/120	29.17 (21.78–37.84)	73/418	3.77 (2.96–4.71)

**Table 3:** PRRSV detection in slaughterhouses and rendering plants in Anhui Province, 2021–2024.

Sampling Period	Site Type	Positive/Total Sites	Site-Level Positivity (%)	Positive/Total Pools	Estimated Individual-Level Positivity, % (95% CI)
Autumn 2021	Slaughterhouse	18/54	33.33	37/327	2.37 (1.67–3.26)
	Rendering plant	8/17	47.06	11/42	5.89 (2.94–10.33)
Spring 2022	Slaughterhouse	21/129	16.28	38/389	2.03 (1.44–2.78)
	Rendering plant	5/20	25.00	5/20	5.59 (1.79–12.64)
Autumn 2022	Slaughterhouse	21/123	17.07	30/389	1.59 (1.08–2.27)
	Rendering plant	5/21	22.73	5/22	5.03 (1.62–11.39)
Spring 2023	Slaughterhouse	36/102	35.30	83/408	4.40 (3.62–5.54)
	Rendering plant	8/21	38.10	8/21	9.10 (3.89–17.43)
Autumn 2023	Slaughterhouse	42/99	42.42	42/394	2.22 (1.61–3.00)
	Rendering plant	7/20	35.00	7/20	8.25 (3.29–16.42)
Spring 2024	Slaughterhouse	35/99	35.35	29/394	1.52 (1.01–2.17)
	Rendering plant	7/21	33.33	7/21	7.79 (3.20–15.52)
Autumn 2024	Slaughterhouse	31/99	31.31	68/397	3.69 (2.87–4.65)
	Rendering plant	4/21	19.05	5/21	5.29 (1.70–11.98)

## Data Availability

The data presented in this study are available from the corresponding author upon reasonable request. The data are not publicly available because they contain herd-level animal disease surveillance information.

## Supplementary Materials

The following supporting information can be downloaded at: https://www.mdpi.com/article/10.3390/ani16172672/s1, Table S1. Information of the PRRSV reference strains used for ORF5-based phylogenetic analysis.

## Abbreviations

PRRSV	Porcine reproductive and respiratory syndrome virus
PRRS	Porcine reproductive and respiratory syndrome
CI	Confidence interval
