# Supplementary material for: Repeated Cross-Sectional Surveillance and ORF5-Based Molecular Epidemiology of Porcine Reproductive and Respiratory Syndrome Virus in Anhui Province, China, 2019–2024
Source: Animals (Basel). 2026 Aug 25;16(17):2672. doi: 10.3390/ani16172672 (PMC13564284; doi:10.3390/ani16172672)
Supplement: Supplementary file 1 [file animals-16-02672-s001.zip › animals-4493584-supplementary.pdf]

Table S1. Information of the PRRSV reference strains used for ORF5-based phylogenetic analysis.

Strain	GenBank accession no.	Year	Area	Strain type
PRRSV/SD17-36/pig/2017	MH121061.1	2017	China	Circulating strain
PRRSV/SD17-38/pig/2017	MH068878.1	2018	China	Circulating strain
PRRSV/WUH5/pig/2015	KU523366.1	2016	China	Circulating strain
PRRSV/CHsx1401/sow/2014	KP861625.1	2014	China	Circulating strain
PRRSV/NADC30/USA/2008	JN654459.1	2008	USA	Circulating strain
PRRSV/HENAN-HEB/pig/China/2012	KJ143621.1	2012	China	Circulating strain
PRRSV/HENAN-XNX/pig/China/2013	KF611905.1	2013	China	Circulating strain
PRRSV/HENXX-8/pig/China/2016	KY041782.1	2016	China	Circulating strain
PRRSV/JL580/China/2013	KR706343.1	2013	China	Circulating strain
PRRSV/SDYG1606/swine/China/2016	KY053458.1	2016	China	Circulating strain
PRRSV/MN184C/USA/2005	EF488739.1	2005	USA	Circulating strain
PRRSV/IA/2014/NADC34/USA/2014	MF326985.1	2014	USA	Circulating strain
PRRSV/QYYZ/pig/China/2011	JQ308798.1	2011	China	Circulating strain
PRRSV/GM2/swine/China/2012	JN662424.1	2012	China	Circulating strain
PRRSV/CH-1a/China/1996	AY032626.1	1996	China	Circulating strain
PRRSV/CH-1R/China/2007	EU807840.1	2007	China	Vaccine strain
PRRSV/SDlz1601/swine/China/2018	KX980393.1	2018	China	Circulating strain
PRRSV/JXA1-P80/China/2009	FJ548853.1	2009	China	Vaccine strain
PRRSV/JXA1/China/2007	EF112445.1	2007	China	Circulating strain

Strain	GenBank accession no.	Year	Area	Strain type
PRRSV/HUN4/China/2007	EF635006.1	2007	China	Circulating strain
PRRSV/R98/China/2006	DQ355796.1	2006	China	Vaccine strain
PRRSV/MLV RespPRRS/Repro	AF159149.1	2000	USA	Vaccine strain
PRRSV/RespPRRS MLV	AF066183.4	2000	China	Vaccine strain
PRRSV/VR-2332/USA/1992	AY150564.1	1992	USA	Circulating strain
PRRSV/BJ-4/2000	AF331831.1	2000	China	Circulating strain
PRRSV/Lelystad virus/Netherlands/1991	M96262.2	1991	Netherla nds	Circulating strain
